# Supplementary material for: Cross-species Standardised Cortico-Subcortical Tractography
Source: bioRxiv. 2025 Jun 11:2025.04.29.651254. Preprint. [Version 3] doi: 10.1101/2025.04.29.651254 (PMC12157545; doi:10.1101/2025.04.29.651254)
Supplement: Supplement 1 [file NIHPP2025.04.29.651254v3-supplement-1.pdf]

bioRxiv preprint doi: https://doi.org/10.1101/2025.04.29.651254; this version posted June 11, 2025. The copyright holder for this preprint (which was not certified by peer review) is the author/funder, who has granted bioRxiv a license to display the preprint in perpetuity. It is made available under aCC-BY 4.0 International license.

Supplementary Materials for Cross-species Standardised Cortico-Subcortical Tractography

Stephania Assimopoulos, Shaun Warrington, Davide Folloni, Katherine Bryant, Wei Tang, Saad Jbabdi, Sarah Heilbronner, Rogier B Mars, Stamatios N Sotiropoulos

*Corresponding author. Email: stamatios.sotiropoulos@nottingham.ac.uk

Original cortico-thalamic XTRACT protocols

For completeness of presenting all the cortico-subcortical protocols, we include here the previously published protocols of thalamic radiations (*31*).

Acoustic Radiation (AR): The acoustic radiation connects the medial geniculate nucleus (MGN) of the thalamus to the auditory cortex. The seed was placed in the transverse temporal gyrus and the target covered the MGN of the thalamus. The exclusion mask consisted of two coronal planes, anterior and posterior to the thalamus, and an axial plane superior to the thalamus. In addition, the exclusion mask contained the brainstem and a horizontal region covering the optic tract.

Anterior Thalamic Radiation (ATR): The anterior thalamic radiation connects the thalamus to the frontal lobe. The seed mask was a coronal mask through the anterior part of the thalamus (*121*), with a coronal target mask at the anterior thalamic peduncle. In addition, the exclusion mask contained an axial plane covering the base of the midbrain, a coronal plane preventing leakage via the posterior thalamic peduncle and a coronal plane preventing leakage via the cingulum. A coronal stop mask covered the posterior part of the thalamus, extending from the base of the midbrain to the callosal sulcus.

Optic Radiation (OR): The optic radiation consists of fibres from the lateral geniculate nucleus (LGN) of the thalamus to the primary visual cortex. The seed was placed in the LGN and the target mask consisted of a coronal plane through the anterior part of the calcarine fissure. Exclusion masks consisted of an axial block of the brainstem, a coronal block of fibres directly posterior to the LGN to select fibres that curl around dorsally, and a coronal plane anterior to the seed to prevent leaking into longitudinal fibres.

Superior Thalamic Radiation (STR): The superior thalamic radiation connects the thalamus to

bioRxiv preprint doi: https://doi.org/10.1101/2025.04.29.651254; this version posted June 11, 2025. The copyright holder for this preprint (which was not certified by peer review) is the author/funder, who has granted bioRxiv a license to display the preprint in perpetuity. It is made available under aCC-BY 4.0 International license.

the pre-/post-central gyrus respectively. The seed was a mask covering the whole thalamus and the target an axial plane covering the superior thalamic peduncle. An axial plane was used as a stop mask ventrally to the thalamus. The exclusion mask included two coronal planes, anterior and posterior to the target, to exclude tracking to the prefrontal cortex and occipital cortex respectively.

bioRxiv preprint doi: https://doi.org/10.1101/2025.04.29.651254; this version posted June 11, 2025. The copyright holder for this preprint (which was not certified by peer review) is the author/funder, who has granted bioRxiv a license to display the preprint in perpetuity. It is made available under aCC-BY 4.0 International license.

***Table S1**: **List of all species-matched (human & macaque) tract protocols**, grouped as cortico-cortical, cortico-subcortical and cerebellar. Columns indicate whether corresponding protocols are bilateral or not and whether they are New, Revised or not changed (Original XTRACT).*

**Cortico-Cortical**	**Abbreviation**	**Bilateral**	**Version**
Arcuate Fasciculus	AF	Yes	Original
Cingulum subsection : Dorsal	CBD	Yes	Original
Cingulum subsection : Peri-genual	CBP	Yes	Original
Cingulum subsection : Temporal	CBT	Yes	Original
Corticospinal Tract	CST	Yes	Original
Frontal Aslant	FA	Yes	Original
Forceps Major	FMA	No	Original
Forceps Minor	FMI	No	Original
Inferior Longitudinal Fasciculus	ILF	Yes	Original
Inferior Fronto-Occipital Fasciculus	IFO	Yes	Original
Middle Longitudinal Fasciculus	$MdLF$	Yes	Original
Superior Longitudinal Fasciculus 1	$SLF1$	Yes	Original
Superior Longitudinal Fasciculus 2	$SLF2$	Yes	Original
Superior Longitudinal Fasciculus 3	$SLF3$	Yes	Original
Vertical Occipital Fasciculus	VOF	Yes	Original
Uncinate Fasciculus	UF	Yes	Revised
**Cortico-Subcortical**			
Acoustic Radiation	AR	Yes	Original
Anterior Thalamic Radiation	ATR	Yes	Original
Optic Radiation	OR	Yes	Original
Superior Thalamic Radiation	STR	Yes	Original
Fornix	FX	Yes	Revised
Anterior Commissure	AC	No	Revised
Amygdalofugal Tract	AMF	Yes	New
Muratoff Bundle/Subcallosal Fasciculus	MB	Yes	New
Striatal Bundle/External Capsule (sensorimotor)	StB_m	Yes	New
Striatal Bundle/External Capsule (frontal)	StB_f	Yes	New
Striatal Bundle/External Capsule (temporal)	StB_t	Yes	New
Striatal Bundle/External Capsule (parietal)	StB_p	Yes	New
Extreme Capsule (frontal)	EmC_f	Yes	New
Extreme Capsule (temporal)	EmC_t	Yes	New
Extreme Capsule (parietal)	EmC_p	Yes	New
**Cerebellar**			
Middle Cerebellar Peduncle	MCP	No	Original

bioRxiv preprint doi: https://doi.org/10.1101/2025.04.29.651254; this version posted June 11, 2025. The copyright holder for this preprint (which was not certified by peer review) is the author/funder, who has granted bioRxiv a license to display the preprint in perpetuity. It is made available under aCC-BY 4.0 International license.

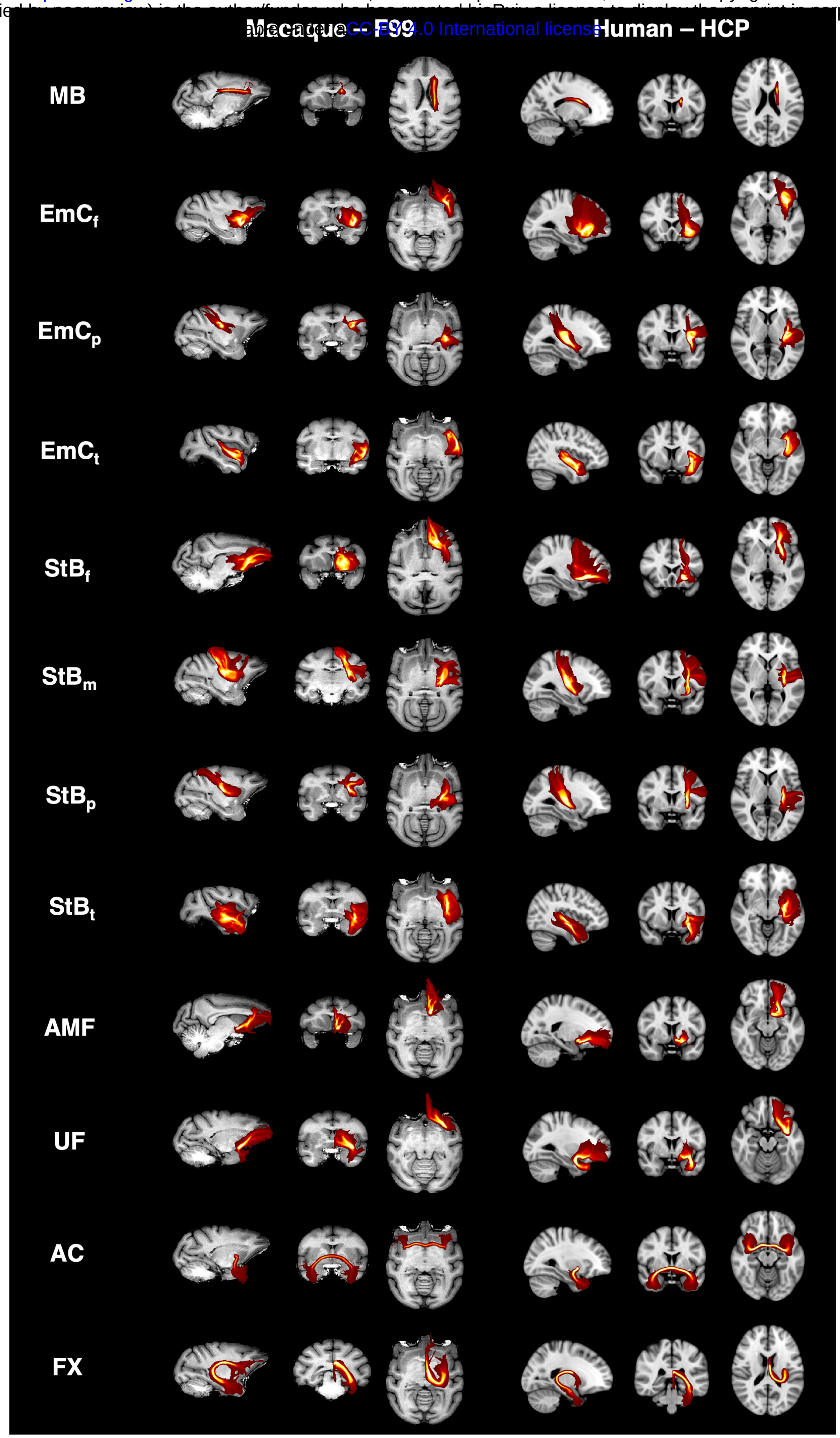

***Figure S1**: **Tract reconstructions using our cortico-subcortical protocols, with good agreement between the macaque and the human.** Maximum intensity projections (MIPs) of the group-averaged path distributions for all developed tractography protocols in the macaque (6 animal average) and human (50 healthy subject average from the Human Connectome Project dataset; HCP). All MIPs are across a window (20% of the field of view) centred at the displayed slices. Thresholded path distributions are displayed with a low threshold of 0.1% (for the EmC parts the 90th percentile was used).*

bioRxiv preprint doi: https://doi.org/10.1101/2025.04.29.651254; this version posted June 11, 2025. The copyright holder for this preprint (which was not certified by peer review) is the author/funder, who has granted bioRxiv a license to display the preprint in perpetuity. It is made available under aCC-BY 4.0 International license.

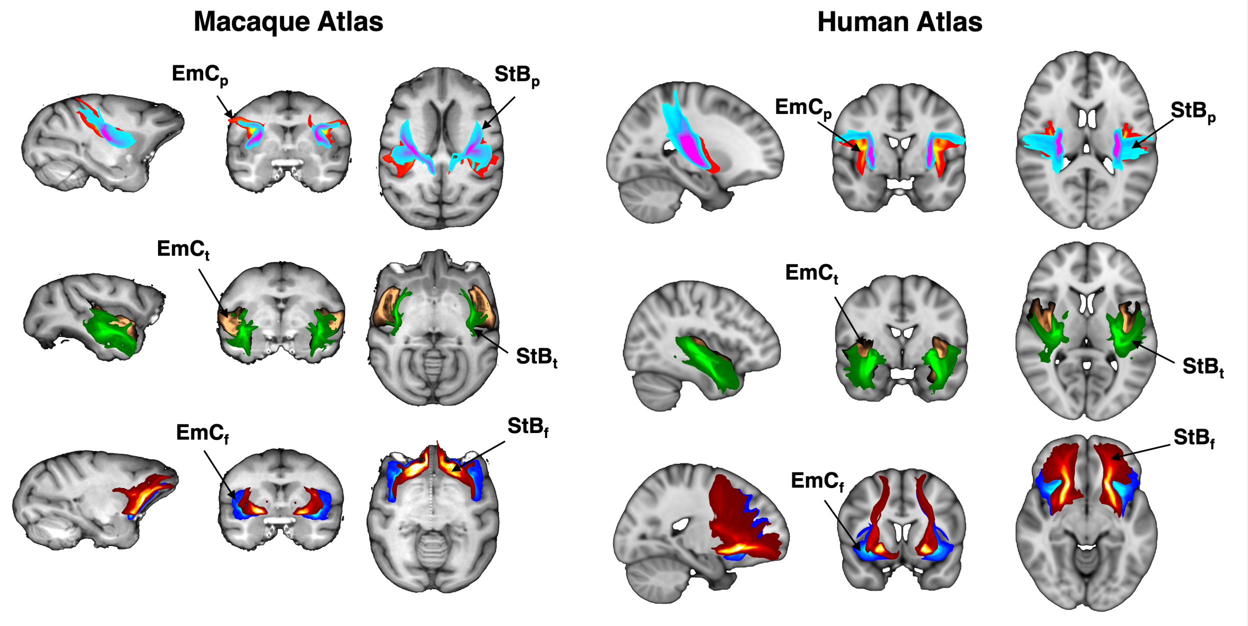

***Figure S2*: Relative positions maintained for all corresponding parts of striatal and extreme capsule bundles, across species.** *Maximum intensity projections (MIPs) of the group-averaged tractography results for corresponding parts of the striatal bundle (StB)/external capsule and the extreme capsule (EmC) in the macaque (6 animal average) and human (50 healthy subject average from the Human Connectome Project dataset; HCP). For each part, StB is more medial and EmC is more lateral (with respect to each other. Tracts considered: frontal, temporal and parietal parts of the anterior limb of the extreme capsule (EmC_f, EmC_t, EmC_p); frontal, temporal and parietal parts of the striatal bundle (StB_f, StB_t, StB_p) (Table 1 in main text).*

bioRxiv preprint doi: https://doi.org/10.1101/2025.04.29.651254; this version posted June 11, 2025. The copyright holder for this preprint (which was not certified by peer review) is the author/funder, who has granted bioRxiv a license to display the preprint in perpetuity. It is made available under aCC-BY 4.0 International license.

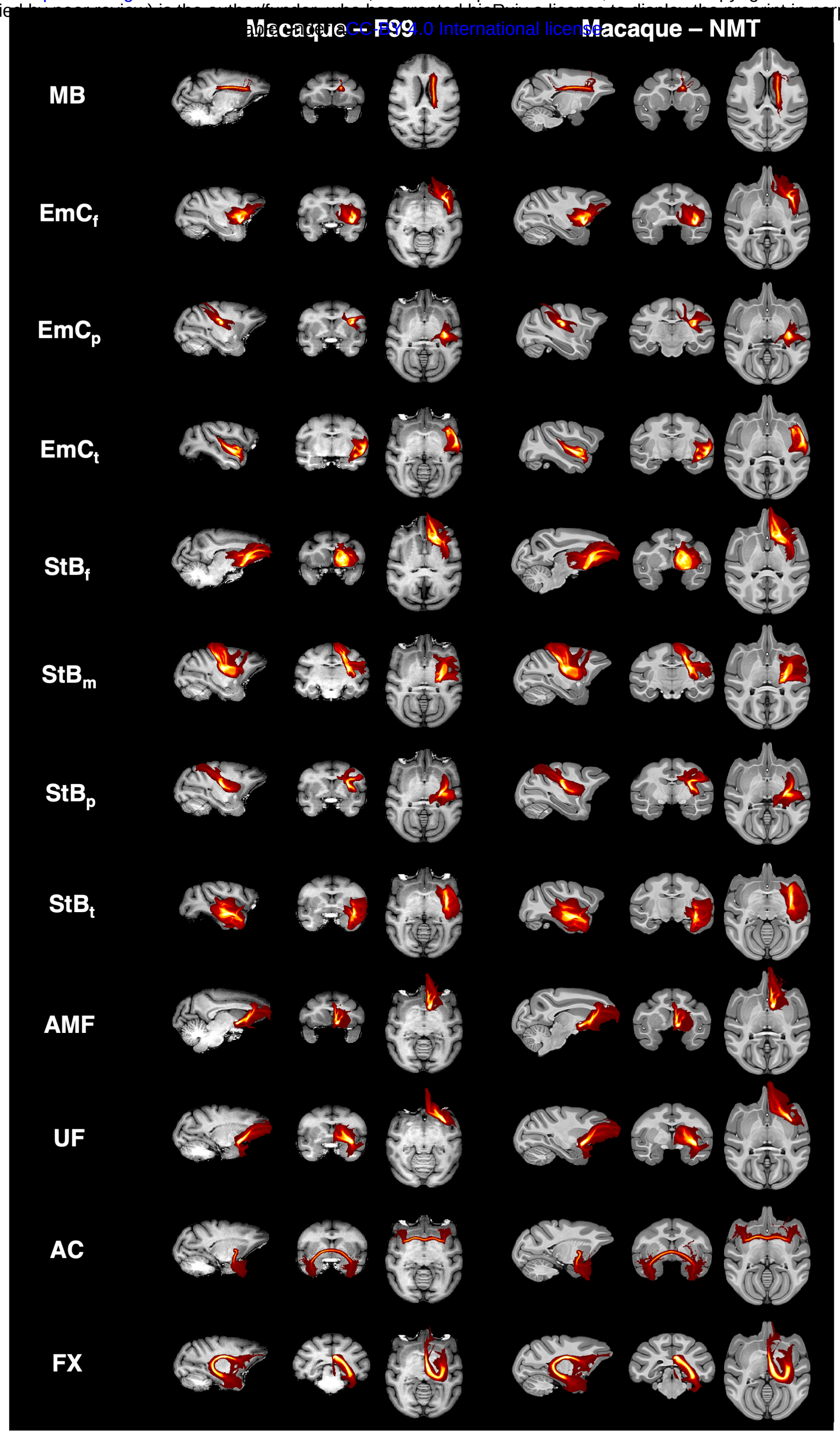

***Figure S3**: **Corresponding tract reconstructions using our macaque cortico-subcortical protocols, between the two macaque standard spaces, F99 and NMT.** Maximum intensity projections (MIPs) of the group-averaged path distributions for all developed tractography results for all developed protocols in the macaque (6 animal average) using protocols in the F99 standard space and protocols in the NMT standard space. All MIPs are across a window (20% of the field of view) centred at the displayed slices. Thresholded path distributions are displayed with a low threshold of 0.1% (for the EmC parts the 90th percentile was used).*

bioRxiv preprint doi: https://doi.org/10.1101/2025.04.29.651254; this version posted June 11, 2025. The copyright holder for this preprint (which was not certified by peer review) is the author/funder, who has granted bioRxiv a license to display the preprint in perpetuity. It is made available under aCC-BY 4.0 International license.

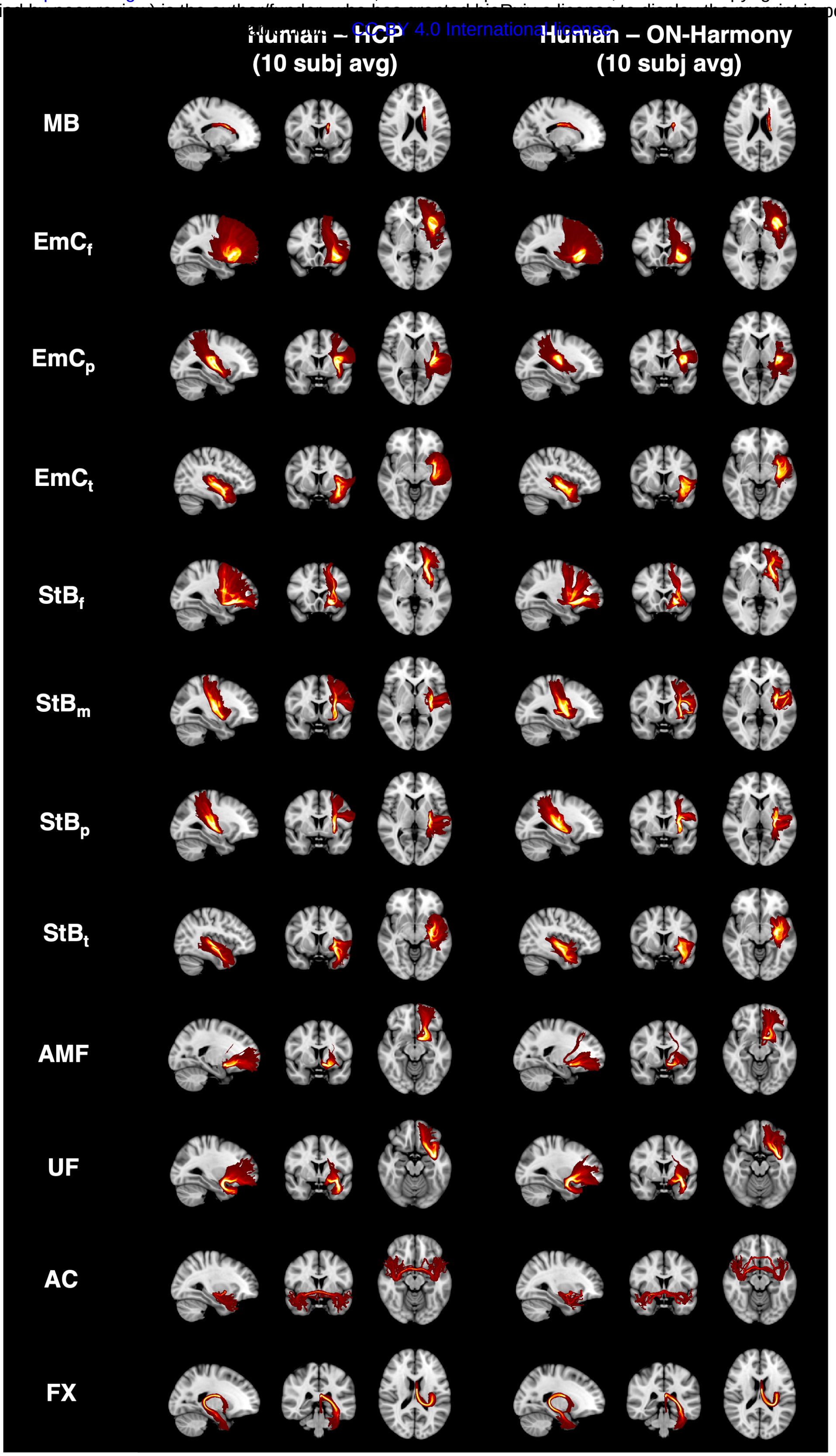

***Figure S4**: Corresponding tract reconstructions using our cortico-subcortical protocols, between different data resolutions (spatial and angular) and acquisition protocols.. Maximum intensity projections (MIPs) of the group-averaged path distributions for all developed tractography results for all developed protocols in 10 Human Connectome Project (HCP) and 10 ON-Harmony (NH; UK-Biobank style acquisition) subjects. All MIPs are across a window (20% of the field of view) centred at the displayed slices. Thresholded path distributions are displayed with a low threshold of 0.1% (for the EmC parts the 90th percentile was used).*
